# VALIDITY EVIDENCE FOR THE MALE DEPRESSION RISK SCALE-22 (MDRS-22) IN YOUNGER AND OLDER ADULT MALES

**DOI:** 10.1093/geroni/igac059.2862

**Published:** 2022-12-20

**Authors:** Montgomery Owsiany, Amy Fiske

**Affiliations:** West Virginia University, Morgantown, West Virginia, United States; West Virginia University, Morgantown, West Virginia, United States

## Abstract

Men are two times less likely to be diagnosed with major depressive disorder (MDD) than women. However, suicide rates are nearly four times higher in men than women, increasing to six times or more when comparing older men to older women. Masculine depression is characterized by symptoms that are not usually assessed by diagnostic criteria for MDD, including drug and alcohol abuse, anger and aggression, and risk-taking. Previous studies have largely neglected to consider the possibility of age-related differences in the presentation of masculine depression. Given research on age-related differences in MDD, there may be age-related differences in the presentation of masculine depression as well. The present study assessed age invariance of the MDRS-22 through a multi-group confirmatory factor analysis (CFA), evaluated validity evidence of the MDRS-22, and tested the MDRS-22’s ability to assess suicidal ideation and behaviors. There was a significant difference between the configural and first-order metric models of the CFA showing that the MDRS-22 was not age invariant (ΔX2 = 451.47, Δdf = 16, p < .001). The MDRS-22 showed convergent validity evidence with assessments of MDD, alignment with masculine norms, suicidal behaviors, problematic alcohol use, and aggression. The MDRS-22 showed concurrent validity evidence with another assessment of masculine depression. Finally, MDRS-22 scores significantly predicted suicide risk above PHQ-9 scores (F(2,440) = 138.774, p < .001, R2 = .385). Overall, the study highlights the importance of screening males for masculine depression. Further research is needed to determine if masculine depression presents differently in younger and older males.

